# Evaluation of the effects of vitamins C and E on experimental orthodontic tooth movement

**DOI:** 10.34172/joddd.2020.0027

**Published:** 2020-06-17

**Authors:** Esra Bolat, Elçin Esenlik, Meral Öncü, Meltem Özgöçmen, Mustafa Cihat Avunduk, Özlem Yüksel

**Affiliations:** ^1^Department of Orthodontics, Faculty of Dentistry, Akdeniz University, Antalya, Turkey; ^2^Department of Histology Embriyology, Faculty of Medicine, Süleyman Demirel University, Isparta, Turkey; ^3^Department of Pathology, Faculty of Meram Medicine, Necmettin Erbakan University, Konya, Turkey; ^4^Şehit Kamil State Hospital, Gaziantep, Turkey

**Keywords:** Histomorphometry, Tooth movement, Vitamin C, Vitamin E

## Abstract

**Background.** This experimental study aimed to assess the effects of Vitamins C and E on orthodontic tooth movement.

**Methods.** Fifty-one male Wistar albino rats were divided into six groups: five appliance groups and one control group. The appliance groups had an orthodontic appliance consisting of a closed-coil spring ligated between the maxillary incisor and maxillary first molar (50 g). Vitamin E and C (150 mg/kg) were injected intraperitoneally per day in the first and second groups, respectively. Vitamins E and C (20 μL) were locally injected into the periodontal gap of the moving teeth in the third and fourth groups, respectively, once every three days. No vitamin was injected in the last (fifth) appliance group.The experimental period was 18 days. Histological and biochemical (alkaline phosphatase, osteocalcin, and NTx levels) evaluations of the samples were performed, and maxillary incisor‒molar distance was measured before and after the experiment.

**Results.** The amount of tooth movement was similar in the appliance groups. All the vitamin groups showed significantly increased osteoblastic activity, while those treated with systemic vitamins exhibited significantly increased numbers of collagen fibers on the tension side compared to the appliance control group (P<0.05).

**Conclusion.** Vitamin C and E supplements positively affected bone formation on the tension side of the teeth during experimental orthodontic tooth movement.

## Introduction

The application of mechanical force to teeth causes orthodontic tooth movement as a result of the biological responses of the surrounding periodontal tissues. Local regeneration involves the resorption of the alveolar bone adjacent to the periodontal ligament in the pressure zone, apposition in the tension zone, and formative and degenerative changes in the periodontal ligament.^1^ It has been well documented that various medications and chemical substances that affect bone metabolism can also affect orthodontic tooth movement.^2^ Vitamins have been among these substances, which act as antioxidants by inhibiting free radicals.^2-5^

Oxygen-derived free radicals are formed by some phagocytes and have been reported to increase in the normal bone formation process, aging, chronic inflammatory diseases, and osteoporosis.^3^ According to previous *in vivo* and *in vitro* studies, free radicals induce bone resorption and osteoclast formation. Administration of antioxidants, such as vitamins, has been shown to be useful in suppressing the damaging effects of free oxygen radicals on cells during bone formation.^3,4^ Vitamin E (α-tocopherol), which is a strong biological antioxidant, prevents an oxidative attack on membrane lipids^5^ and has also been shown to suppress the production of certain pro-inflammatory mediators that have been related to increased bone loss.^6^ In addition, α-tocopherol was found to improve the calcium content and mechanical properties of bone tissue in a previous study.^7^ Similarly, vitamin C (ascorbic acid) has also been shown to neutralize the effects of free radicals on body fluids and reverse free radical-mediated damage on a cellular level.^8^ A relationship has also been reported between collagen synthesis and ascorbic acid. Collagen fibers, which contain proline and hydroxyproline, is a major constituent of teeth and their surrounding supportive structures.^9^ Although previous studies have reported the effects of vitamins C and E on bone metabolism, there remains a gap in knowledge regarding the effects of these vitamins on orthodontic tooth movement. Therefore, in the present study, we aimed to assess the systemic and local effects of vitamins E and C on orthodontic tooth movement via histological and biochemical methods.

## Methods

Our study plan was approved by the Suleyman Demirel University Medical Faculty Ethics Committee for Experimental Animals before the study. Six-to-eight-week-old Wistar Albino rats weighing 120‒180 g were included in the study. There were ten Wistar Albino rats in each experimental group, with six rats in the control group. They were exposed to standard 12-hour light/dark cycles at a constant temperature of 24°C and fed *ad libitum* with water and ground rat food. The rat food was softened with water to avoid potential appliance breakage.

### Experimental design and protocol

The rats were randomly assigned to six groups; the control group included six rats, while each experimental group consisted of 10 rats (as suggested by the Ethics Committee). Five animals were lost during the experiments due to feeding problems; hence, the study was finalized with 51 rats. The groups and experiment protocols are described below:

**Group I** (systemic vitamin E group [**SE**]) (n=9). The orthodontic appliance was applied, along with daily intraperitoneal injections with 150 mg/kg of vitamin E (Evigen (dl-Alfa Tokoferol Asetat).

**Group II** (systemic vitamin C group [**SC**]) (n=10). The orthodontic appliance was applied, along with daily intraperitoneal injections with 150 mg/kg of vitamin C (Redoxon, Bayer, Leverkusen).

**Group III** (local vitamin E group [**LE**]) (n=9). The orthodontic appliance was applied, along with local injections of 20 µL of vitamin E once every three days.

**Group IV** (local vitamin C group [**LC**]) (n=8). The orthodontic appliance was applied, along with local injections of 20 µL of vitamin C once every three days.

**Group V** (appliance control group [**AC**]) (n=9). The rats did not receive any vitamin injections. This group was included to examine the amount of orthodontic tooth movement and histological changes caused by mechanical force alone.

**Group VI** (baseline control group [**BC**]) (n=6). This group served as a baseline control, and the sample size conformed to that accepted by the Ethics Committee. The rats received no treatment and were kept under the same conditions as the experimental groups and monitored during the experimental period. The histological properties of non-treated rat periodontium and biochemical properties of rat serum were compared with those of the experimental groups.

**Figure 1:**
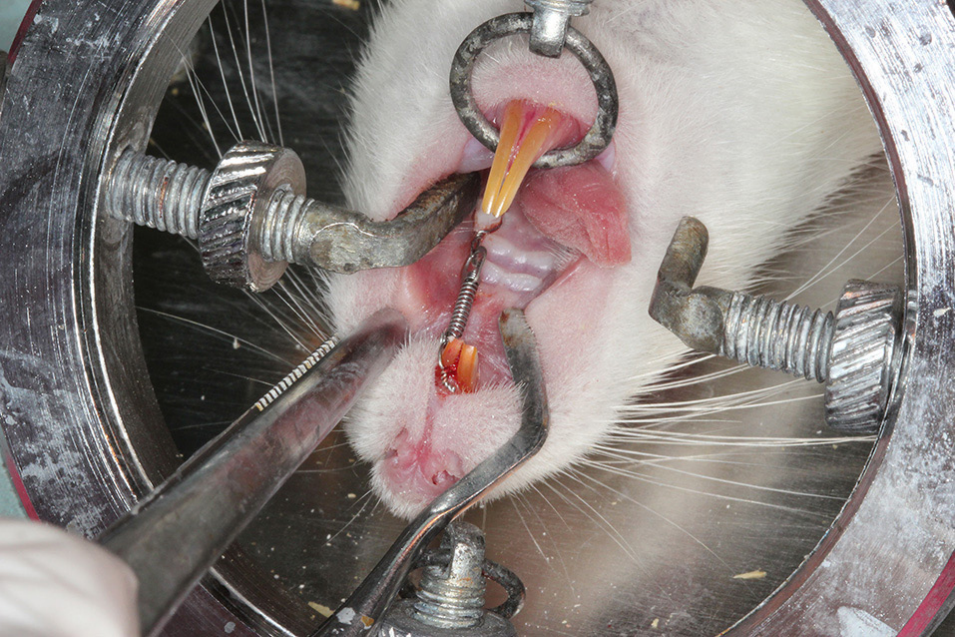
The orthodontic appliance consisting of a closed-coil spring ligated between the maxillary incisor and maxillary first molar.

The experimental period was 18 days. Local injections were performed with a microsyringe (Hamilton Company, Nevada, USA) into the periodontal area of the maxillary first molar under general anesthesia. The rats were held in position with an animal-holding board during these applications (Figure 1).

### Application of the orthodontic appliance

**Figure 2:**
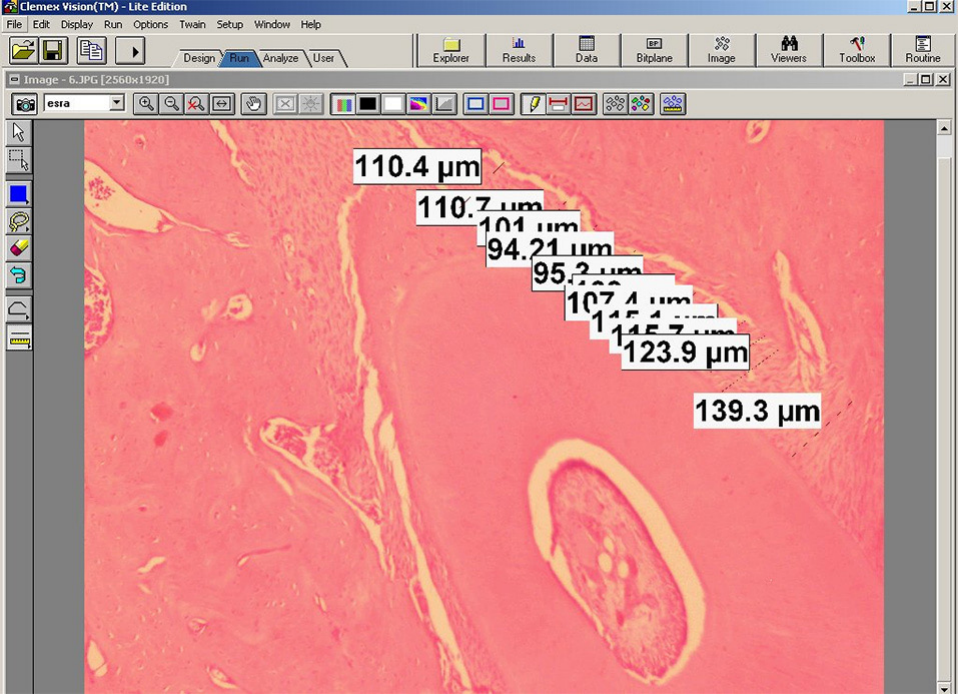
Measurement of periodontal gap on mesial and distal roots of the first molarat 10 different points (Group II) (H-E, 40X)

Experimental tooth movement was induced using a modification of the method described by Hashimoto et al^9^ with a closed-coil spring (6 mm) (American Orthodontics, Sheboygan, USA) with eyelet-like attachments ligated to the maxillary first molar by a stainless-steel ligature wire (G&H Wire Company, USA). The other side of the coil spring was also ligated, with grooves in the maxillary incisors drilled just above the gingival papilla using the same ligature wire (Figure 2). The ligature wire around the incisor was attached with light-cured composite resin (Ormco LCBC; Glendora, CA, USA). A closed coil spring with a force of 50 g was applied to the upper first molar to move it mesially.^10,11^

### Orthodontic tooth movement measurements

Since the first molars in rats drift distally while the incisors continue to erupt, the split-mouth design was the preferred method for determining the amount of tooth movement.^12^ Although the orthodontic appliance was inserted on the left side in all the rats, the distance between the palatal surface of the maxillary incisor and the mesial surface of the maxillary first molar was measured on both sides using a digital caliper intraorally. These measurements were performed by two different researchers before the insertion of the appliance (T_0_) and at the end of the experimental period (T_1_). The inter-class coefficient between the two researchers’ measurements was 0.995‒0.999.

After the experimental period, phlebotomy (via the inferior vena cava, 8 mL) was performed for biochemical analysis. Thereafter, the rats were euthanized, and their upper left first molars, along with the alveolar bone, were dissected for histological examinations.

### Histological preparation and histomorphometric analysis

The tissue samples were fixed in 10% neutral-buffered formalin solution. After 48 h of fixation, the specimens were placed in 10% EDTA for decalcification.^10^ After decalcification, the tissue samples were examined by routine light microscopic techniques. The paraffin-embedded blocks were cut to 4‒5-µm-thick sections, which were stained with hematoxylin–eosin (HE) and Masson’s trichrome. The stained sections were then examined under a light microscope (Nikon Eclipse E400). All the histopathological evaluations were performed in a double-blinded plan.

For each specimen, the same area was imaged using a Nikon Coolpix 5000 camera (Nikon, Tokyo, Japan). Photographs of the Nikon micrometer microscope slide (MBM11100 Stage Micrometer Type A) was also taken during the procedure. All the images were then transferred onto a computer, and the mesial and distal halves of the roots were measured using Clemex Vision Lite 3.5 (Clemex Technologies, Quebec, Canada). The length was calibrated by comparing the photograph of the specimen with the photograph of the Nikon micrometer microscope slide (MBM11100; Nikon Corp., Tokyo, Japan), which was taken under the same magnification.

**Figure 3:**
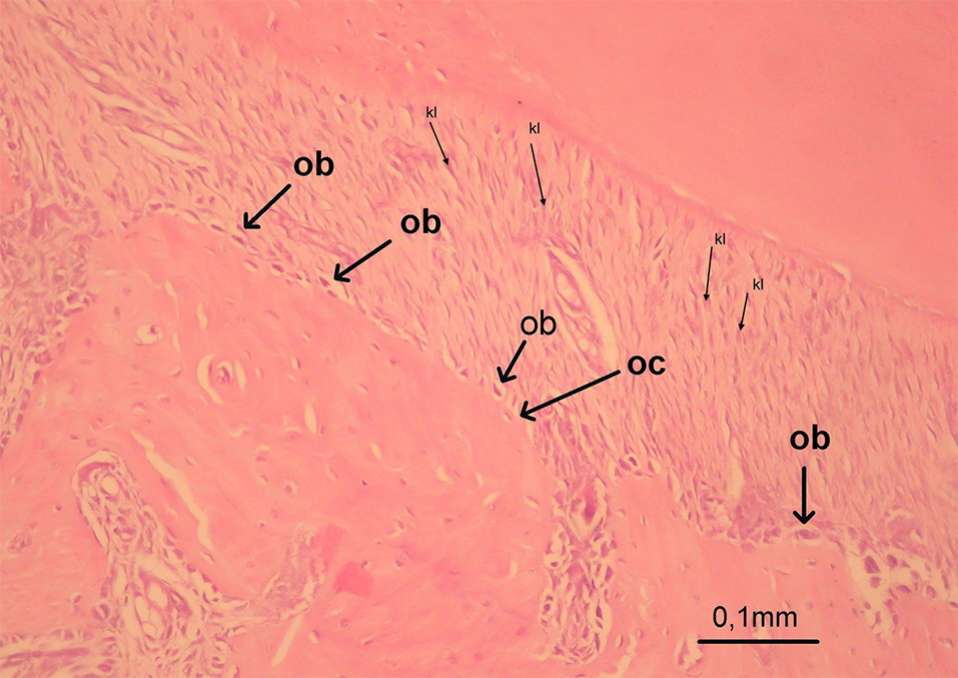
Osteoblast and osteoclast cells at the mesial and distal halves of the roots (Group III) (H-E, 100X)

**Figure 4:**
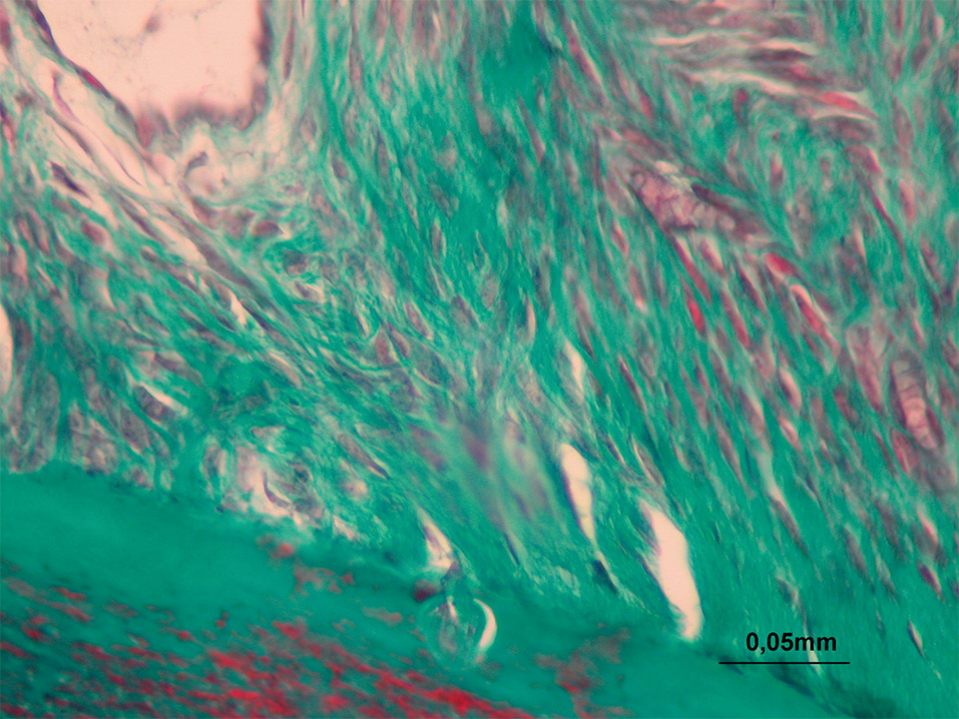
Collagen fibers at the mesial and distal halves of the roots (Group V) (M-T, 400X)

The periodontal gap (the distance between the outer border of the tooth root and the alveolar bone) on the mesial and distal roots of the first molar was measured at 10 different points, and the mean values were recorded for the mesial and distal surfaces. These measurements were repeated for each visible root of the first molar on the experiment side (upper left), and the means were calculated for each tooth (Figure 3). 14055878 µm² areas were designated with the same image analysis system at the mesial and distal halves of the roots. Thereafter, collagen fibers, osteoblasts, and osteoclasts were marked using the same program in the 14055878 µm² areas (Figures 4). Damaged cells were not evaluated. The marked cells were automatically counted with the image analysis system. All the histomorphometric measurements were performed using the Clemex Vision Lite 3.5 Image Analysis Program.

### Biochemical analyses

Approximately 8 mL of venous blood was collected from the inferior vena cava for biochemical analyses and stored at 4°C, followed by centrifugation at 3000 rpm for 8 minutes. Serum samples were kept at -80°C until measurements. Serum collagen type I, osteocalcin/Bone Gla Protein (OC/BGP), and serum alkaline phosphatase and NTx (N-telopeptide of type I collagen) levels were determined using commercial kits (TSZ ELISA KIT, Framingham, USA) via ELISA on Organon Teknika Microwell System Reader 530 (Austria).

### Statistical analyses

The homogeneity of the variant-covariant matrix was evaluated via Box’s M test for all data first. Normality of distribution was investigated with the Anderson-Darling test, and parametric tests were performed.

Differences between the vitamin groups were analyzed with repeated-measures ANOVA. The differences between the vitamin groups and the control groups (appliance control group and the baseline control group) were analyzed with Dunnett’s test. Mann-Whitney U test was used for osteocalcin and NTx parameters. Vitamins for each application and application for each vitamin were separately compared. P<0.05 was accepted as an indicator of statistical significance.

## Results

### Orthodontic tooth movement measurements

In all the groups with the orthodontic appliance, there were significant decreases in the distance between the molar and incisor teeth on both sides (with or without appliance). However, no significant difference was observed in the amount of orthodontic tooth movement between the groups (Table 1).

**Table 1:** Distances between incisor and molar teeth on the right (control) and left (movement) sides at the beginning and the end of the experiment

	**Incisor-Molar Distances (mm)**				**Movement** **(mm)**
	**T1 ($\bar{x}\pm S\bar{x}$)**		**T2 ($\bar{x}\pm S\bar{x}$)**		**T2-T1**
**Groups**	**Right**	**Left**	**Right**	**Left**	**Left**
**Group I (SE)**	11.84±0.31a*a* *	12.15±0.39a*a* *	11.22±0.29b*b*	10.04±0.34b*b*	1.49
**Group II (SC)**	11.79±0.53a*a*	12.10±0.51a*a*	10.91±0.35b*b*	9.95±0.36b*b*	1.27
**Group III (LE)**	11.97±0.30a*a*	12.01±0.14a*a*	11.17±0.58b*b*	10.00±0.53b*b*	1.21
**Group IV (LC)**	11.68±0.46a*a*	11.87±0.41a*a*	11.37±0.41b*b*	9.85±0.48b*b*	1.71
**Group V (AC)**	11.82±0.38a*a*	12.29±0.61a*a*	10.94±0.60b*b*	9.99±0.44b*b*	1.43
**Group VI (BC)**	12.23±0.44a*a*	12.17±0.40a*a*	12.04±0.51b*b*	11.99±0.40a*a*	0.00

*Small letter indicates differences between groups, bold, italic small letters indicates differences between the beginning and the end of the experiment (Dunnet-t test) (P<0.05).

### Histomorphometric findings

After experimental orthodontic tooth movement, osteoblast counts on the distal side were significantly higher in all the appliance groups. Additionally, osteoblast numbers of the appliance groups were significantly higher in all the vitamin groups than the appliance control group (P<0.05). No difference was observed between the BC and AC groups on the mesial side, while significant increases were observed on the distal side of all the appliance groups (P<0.05; Table 2).

**Table 2:** Osteoblast and osteoclast numbers of left first molar teeth on the mesial and distal sides in experiment and control groups

	**Osteoblast Counts (number)**		**Osteoclast (number)**	
**Groups**	**Mesial($\bar{x}\pm S\bar{x}$)**	**Distal( ($\bar{x}\pm S\bar{x}$)**	**Mesial($\bar{x}\pm S\bar{x}$)**	**Distal( $\bar{x}\pm S\bar{x}$)**
**Group I (SE)**	13.75±2.60Ba*	19.87±3.48Aa	1.88±0.83Aa	1.00±0.53Ba
**Group II (SC)**	13.55±3.08Ba	18.66±2.12Aa	2.22±0.67Aa	0.89±0.60Ba
**Group III (LE)**	13.33±3.01Ba	19.50±2.73Aa	2.00±0.63Aa	0.83±0.75Ba
**Group IV (LC)**	12.83±2.22Ba	19.83±0.98Aa	1.83±0.75Aa	0.83±0.75Ba
**Group V (AC)**	11.80±1.75Ba	13.80±2.04Ab	2.10±0.88Aa	1.10±0.88Ba
**Group VI (BC)**	12.16±0.75Ba	12.16±1.60Bc	0.83±0.40Ab	0.67±0.52Aa

*Capital letters indicates differences between directions, small letters indicates differences between groups (P<0.05).

At the end of the experiment, osteoclast counts were significantly higher on the mesial sides of all the appliance groups (P<0.05), while no significant difference was found between the appliance groups. No difference was observed in osteoclast counts between the BC and appliance groups on the distal side, while there were significant differences on the mesial side (Table 2). Similarly, the periodontal gap was significantly wider on the distal side in all the appliance groups than the BC group (P<0.05). However, no difference was found between the appliance groups (Table 3).

Increased collagen fibers were observed on the distal side in all the appliance groups (P<0.05). When the appliance control groups were compared with the vitamin groups, collagen fibers were found to be significantly more numerous in vitamin E and C groups (P<0.05, Table 3).

**Table 3:** Periodontal gap diameters and collagen fiber counts of left first molar teeth on the mesial and distal sides in experiment and control groups

	**Periodontal Gap (µm)**		**Collagen Fiber Counts**	
**Groups**	**Mesial($\bar{x}\pm S\bar{x}$)**	**Distal($\bar{x}\pm S\bar{x}$)**	**Mesial($\bar{x}\pm S\bar{x}$)**	**Distal($\bar{x}\pm S\bar{x}$)**
**Group I (SE)**	108.35±14.07Bb	147.37±29.34Aa	2.13±0.64Ba	4.00±0.76Aa
**Group II (SC)**	105.01±7.40Bb	140.03±6.41Aa	2.00±0.50Ba	3.89±1.05Aa
**Group III (LE)**	105.82±4.23Bb	136.75±14.84Aa	2.17±0.40Ba	3.50±0.84Ab
**Group IV (LC)**	104.73±6.76Bb	138.88±6.86Aa	2.00±0.63Ba	3.33±0.58Ab
**Group V (AC)**	103.81±8.86Bb	151.79±20.61Aa	1.50±0.53Ba	2.90±0.57Ab
**Group VI (BC)**	129.45±12.23Ba	127.42±10.61Ba	1.83±0.75Ba	1.83±0.75Bc

*Capital letters indicates differences between directions, small letters indicates differences between groups (Dunnet-t test) (P<0.05).

### Biochemical findings

No significant differences in the alkaline phosphatase levels were observed between the experimental and control groups (Table 4). Osteocalcin levels were significantly higher in the LE and LC groups than the SE and SC groups. A comparison between the application groups and the BC group separately in osteocalcin levels showed significantly higher levels in the LC group.

**Table 4:** Alkaline phosphatase, osteocalcin and NTx levels on experiment and control groups (T1)

**Groups**	**Alkaline phosphatase ( mIU /mL) ($\bar{x}\pm S\bar{x}$)**	**Osteocalcin Levels ( pg /mL)** **($\bar{x}\pm S\bar{x}$)**	**NTx Levels (ng/L)** **($\bar{x}\pm S\bar{x}$)**
**Group I (SE)**	67.75±66.64a*	46.83±11.28b	127.08± 55.37a
**Group II (SC)**	49.70±27.84a	44.19±5.29b	91.67±19.24a
**Group III (LE)**	58.63±35.54a	86.04±10.55b	65.59±18.26b
**Group IV (LC)**	70.31±33.29a	204.46±79.61a	71.29±23.21b
**Group V (AC)**	103.92±46.78a	56.86±20.14b	106.70±18.03a
**Group VI (BC)**	36.16±29.28a	41.73±18.67b	119.08±20.82a

*Small letters indicates significant differences between groups (Variant analyses, Dunnet-t test).

NTx levels were significantly lower in the LE group compared to the SE and appliance control groups. NTx levels of LC and LE groups were significantly lower than those in the BC group (Table 4).

## Discussion

Previously, researchers focused on various methods to shorten the orthodontic treatment duration while avoiding potential side effects resulting from the orthodontic mechanics and providing stable results.^13^ For this purpose, procedures including physicomechanical stimulations,^14^ surgically assisted tooth movement,^15,16^ and local and systemic chemical agents, including vitamins^17^ that affect bone turnover, have been reported in the literature. Vitamins with antioxidant effects were shown to eliminate free radicals, which adversely affect the healing process of bone fractures and other wounds, thereby aiding the healing process.^4,17,18^ Antioxidants were also used in experimental studies to accelerate bone formation, and decrease the time required for bone healing in distraction osteogenesis and rapid maxillary expansion procedures.^3,4,17,19^ Most of these studies evaluated vitamin D metabolites. To the best of our knowledge, the present report is the first experimental study that evaluated the effects of vitamins C and E on orthodontic tooth movement. This study revealed that vitamins C and E did not affect the rate of orthodontic tooth movement; however, they significantly increased bone formation on the tension side.

Rats have been considered favorable animals for studying bone remodeling in response to mechanical forces^20^ and have frequently been used in many studies on experimental orthodontic tooth movement.^21,22^ In the present study, the distance between incisor and molar teeth was measured at the beginning and end of the treatment to evaluate the amount of orthodontic tooth movement. Distances on the left (movement) side significantly decreased in all the experimental groups (P<0.05). Interestingly, distances on the right (control) side also significantly decreased in all the appliance groups. This can be explained by the pulling effect of ligating wire on the left side, affecting the right incisors, pulling them distally.^12^ Furthermore, it was previously found that in rats, the molars drift distally over time, and this phenomenon can impact the amount of tooth movement.^12^ Therefore, a control group without any appliance was used in the present study.

Alveolar bone remodeling is one of the most important steps in orthodontic tooth movement. According to histomorphometric analyses, no differences were observed in osteoclast counts, which are the primary mediators of bone resorption between the mesial and distal sides of the BC group. However, the osteoclast counts were significantly higher on the mesial sides in all the experiment groups. This was compatible with previous studies that reported bone resorption in the direction of movement.^23^ The present study showed that the administration of vitamins C and E did not affect bone resorption on the pressure side, while it increased bone formation on the tension side. Furthermore, the osteoblast counts on the tension side were significantly higher in all the vitamin groups compared to the AC group. This finding suggested that vitamins C and E induced bone formation in the tension area. Uysal et al^17^ injected α-tocopherol into the expansion area of orthopedically enlarged inter-pre-maxillary suture and found that early α-tocopherol injection stimulates bone formation and shortens the retention period. Similarly, systemic vitamin C application in rats resulted in a more stable and wider callus and increased the newly-formed bone tissue compared to the control group at the maxillary expansion area in another study.^24^

Osteoblast and osteoclast functions can be determined by various biochemical evaluations.^25^ Garnero and Delmas reported that bone resorption and formation rates could be evaluated by analyzing bone matrix components or osteoblastic and osteoclastic enzyme activities in blood or urine samples.^26^ In the present study, osteoblastic and osteoclastic activities were evaluated biochemically using blood samples. Osteocalcin and alkaline phosphatase levels were assessed for osteoblastic activity, while osteoclastic activity was assessed with NTx level, which is one of the best resorption markers and a collagen metabolite. No significant differences were found in serum alkaline phosphatase levels between the experimental and control groups. In addition, osteocalcin and NTx parameters did not show consistent results with the histomorphometric measurements in our study. Similarly, King and Keeling reported inconsistent results in serum alkaline phosphatase levels and histomorphometric findings during experimental tooth movement.^27,28^ Previous studies indicated that biochemical analyses could not always be consistent in orthodontic tooth movement studies since systemic biochemical values could be affected by numerous parameters.^27,29^

It was previously reported that the periodontal gap width in rat molars remained stable by maintaining the thickness of the alveolar structure naturally.^23^ Confirming this, in the present study, no difference was found in the periodontal gap width in the BC group. However, periodontal gap widths were narrower on the pressure sides of all the appliance groups, but no significant difference was found between the groups. Periodontal gap and ligaments are supposed to be reorganized by fibroblast-mediated collagen synthesis following orthodontic tooth movement.^25,29^ Consequently, localized bone and intense collagen remodeling are expected along the periodontal ligament.^25,30^ However, there are few studies in the literature to have evaluated the relationship between the number of collagen fibers and orthodontic tooth movement. Therefore, two different slices were obtained to evaluate collagen fibers with Masson’s trichrome staining in addition to HE staining in the present study.^31^ Consistent with previous studies, our findings showed a significant increase in the number of collagen fibers in the tension area of all the appliance groups. Vitamin C has also been known to be directly related to collagen synthesis in previous experimental studies.^9^ In a study conducted on vitamin C-deficient pigs, it was reported that the interruption of normal collagen synthesis was shown during periodontal ligament organization during tooth movement.^9^

It can be considered that the increased collagen synthesis around the periodontal area might provide better reorganization of the ligaments and more stable orthodontic tooth movement results based on previous and the present study.

## Conclusion

In this experimental study, the application of systemic or local vitamin C and E did not affect the orthodontic tooth movement rate. However, osteoblastic activity was higher at the tension side in all the vitamin groups. Collagen fibers were also found to be more numerous in the systemic vitamin C and E groups at both tension and pressure sides compared to the appliance group without vitamin following orthodontic tooth movement.

Administration of vitamins C and E during orthodontic tooth movement might be helpful in shortening the retention period and decreasing the risk of relapse due to the demonstrated positive effects on bone remodeling at the tension area. Further studies are needed to evaluate the possible side effects.

## Conflict of Interests

The authors declare no conflict(s) of interest related to the publication of this work.

## Authors’ Contributions

EB and EE did experimental design, evaluating the results, writing and editing the manuscript. MON did histological evaluations and MOZ prepared the histological samples and evaluations of the results.MC and OC did the histomorphometric and biochemical analysis. All authors read and approved the final manuscript.

## Acknowledgments

Not applicable.

## Funding

This research was supported by Center for Scientific Investigation Projects of Süleyman Demirel University, Isparta, Turkey (Project Number: 3199-D1-12).

## Competing interests

The authors declare no competing interests with regards to the authorship and/or publication of this article.

## Ethics Approval

Our study plan was approved by the Süleyman Demirel University Medical Faculty Ethics Committee for Experimental Animals before commencement of the study. Ethical approval form was uploaded separately.
